# Iatrogenic bile duct injury during cholecystectomy presenting after 11 years as a biliary stricture: a case report

**DOI:** 10.1186/s13256-019-2322-2

**Published:** 2020-01-22

**Authors:** Dhruba Narayan Sah, Ramesh Singh Bhandari

**Affiliations:** 1 Institute of Medicine, TU Teaching Hospital, Kathmandu, 00977 Nepal; 2 Department of Gastrointestinal and General Surgery, Maharajgunj, P.O. 1524, Kathmandu, Nepal

**Keywords:** Benign biliary stricture (BBS), Hepaticojejunostomy (HJ), Bile duct injury (BDI), Tribhuvan University Teaching Hospital (TUTH)

## Abstract

**Background:**

Benign biliary stricture is an infrequent condition and the majority occur following cholecystectomy. This case report highlights the occurrence of such a stricture 11 years after cholecystectomy without development of biliary cirrhosis.

**Case presentation:**

Our patient was a 55-year-old Nepalese woman who presented to our hospital with cholangitis of 1-month duration and a history of cholecystectomy 11 years ago. A diagnosis of benign biliary stricture without features of biliary cirrhosis was made, and the patient was successfully managed with a multidisciplinary approach.

**Conclusion:**

Benign biliary stricture can present even decades after cholecystectomy. Roux-en-Y hepaticojejunostomy is the treatment of choice, and a long-term favorable outcome can be expected.

## Introduction

Iatrogenic injury following cholecystectomy is the most common cause of benign biliary stricture (BBS) [1]. Biliary stricture can present after months to years. Biliary strictures may present with pain, jaundice, cholangitis, pruritus, or only with alteration of liver function tests. These injuries are associated with high morbidity, mortality, and prolonged hospitalization [2]. If managed inadequately, they can result in life-threatening complications such as cholangitis, secondary biliary cirrhosis (SBC), and portal hypertension. Surgical procedures should be performed with a multidisciplinary approach in collaboration with skilled and experienced hepatobiliary surgeons, interventional radiologists, and gastroenterologists at a tertiary referral center [3]. If left untreated, they can lead to SBC [4]. The aim of surgical management of biliary strictures is to alleviate the obstruction, prevent secondary hepatocellular damage, and prevent restenosis. The objective of this case report is to explain that biliary stricture can present after decades and without development of portal hypertension or SBC.

## Case presentation

**Fig. 1:**
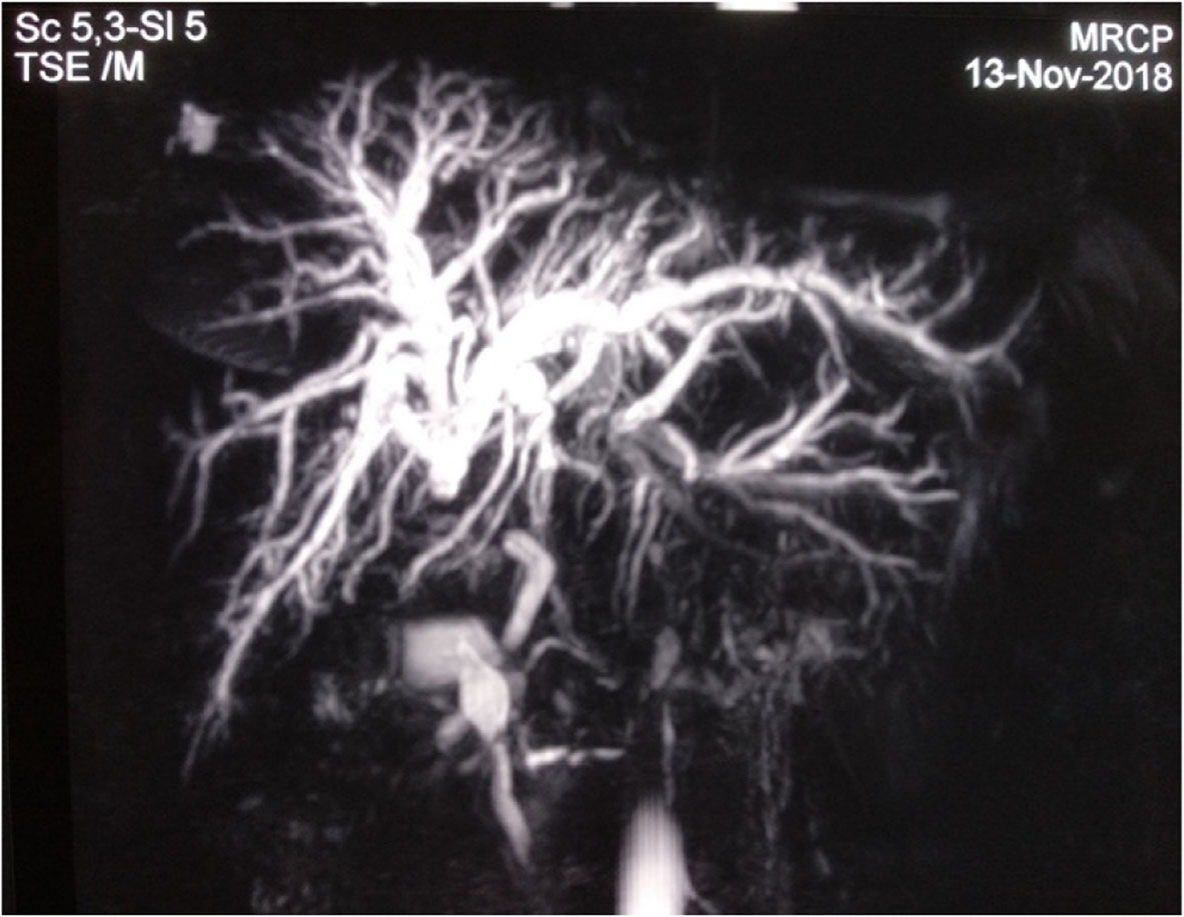
Severe stricture at common hepatic duct (stump 1.4 cm below hilum) with grade II dilated intrahepatic biliary ducts

A 55-year-old Nepalese woman was admitted to our hospital with cholangitis. She had undergone surgery 11 years earlier for cholelithiasis, when a laparoscopic procedure was converted to an open one. Her postoperative course was complicated by biliary peritonitis, for which she underwent reoperation. Eleven years later, the patient presented with fever as well as pain over her right upper quadrant with jaundice for the last 4 weeks. Initially, she was admitted and managed conservatively with symptomatic relief. On further evaluation, she was icteric with moderate dilation of intrahepatic biliary ducts (IHBDs) and the common hepatic duct (CHD) with nonvisualization of the common bile duct (CBD) by ultrasonography. Magnetic resonance cholangiopancreatography (MRCP) revealed severe stricture at the CHD (CHD stump length 1.4 cm below hilar confluence) with grade II dilated IHBDs (Fig. 1). Endoscopic retrograde cholangiopancreatography (ERCP) revealed a nondilated CBD with complete cutoff and no passage of contrast or guidewire above the stricture level, likely representing CHD stricture.

**Fig. 2:**
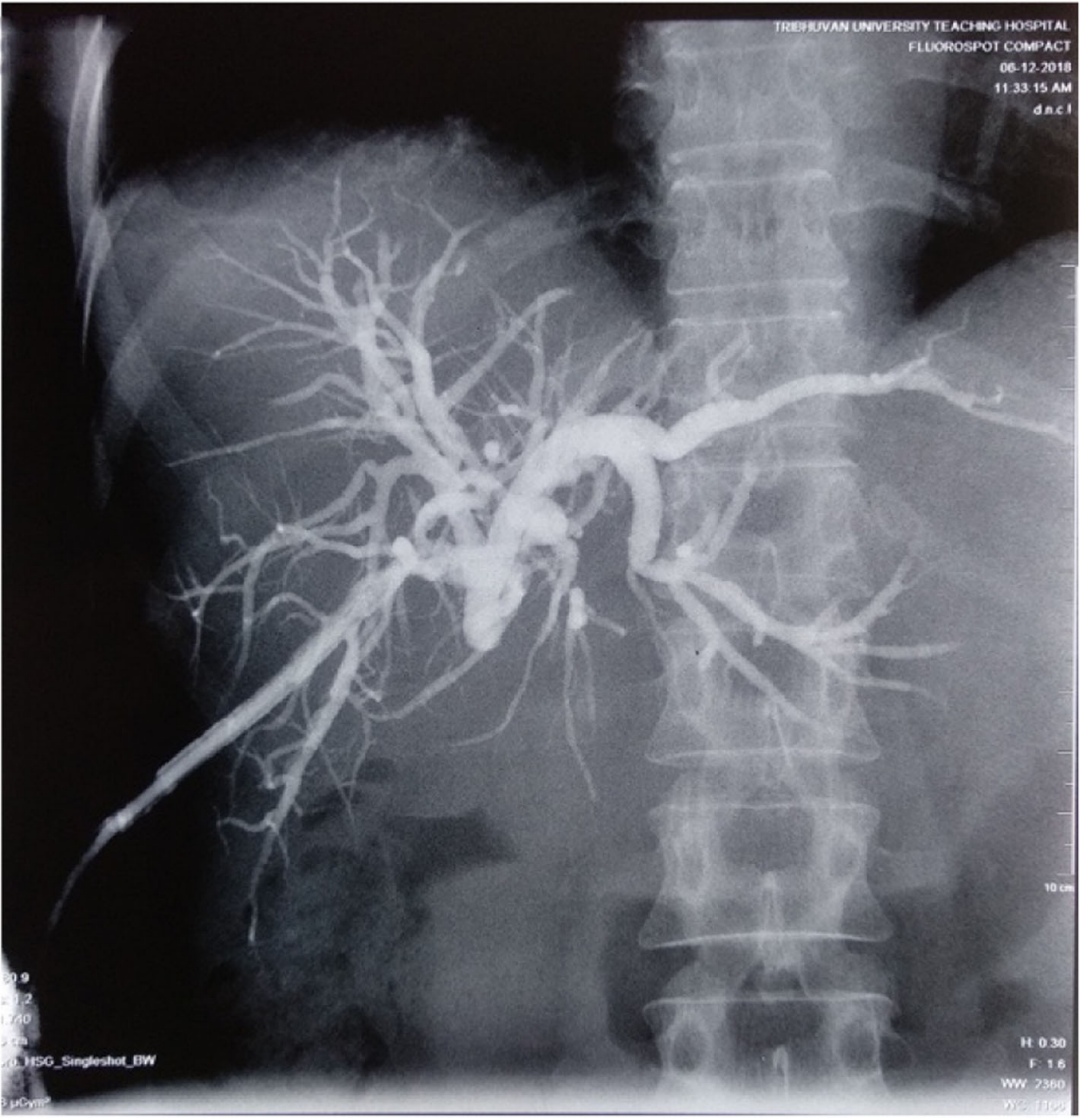
Preoperative percutaneous cholangiogram showing dilated intrahepatic biliary duct and common hepatic duct with complete cutoff

The patient underwent percutaneous biliary drainage (Fig. 2), following which full symptomatic relief was achieved with resolution of cholangitis, including normal biochemical and hematological parameters (except alkaline phosphatase of 457 U/L and cancer antigen 19.9 of 86.7 U/mL). A diagnosis of Bismuth type II biliary stricture (Strasberg bile duct injury E2) was made, and bilioenteric anastomosis was planned.

**Fig. 3:**
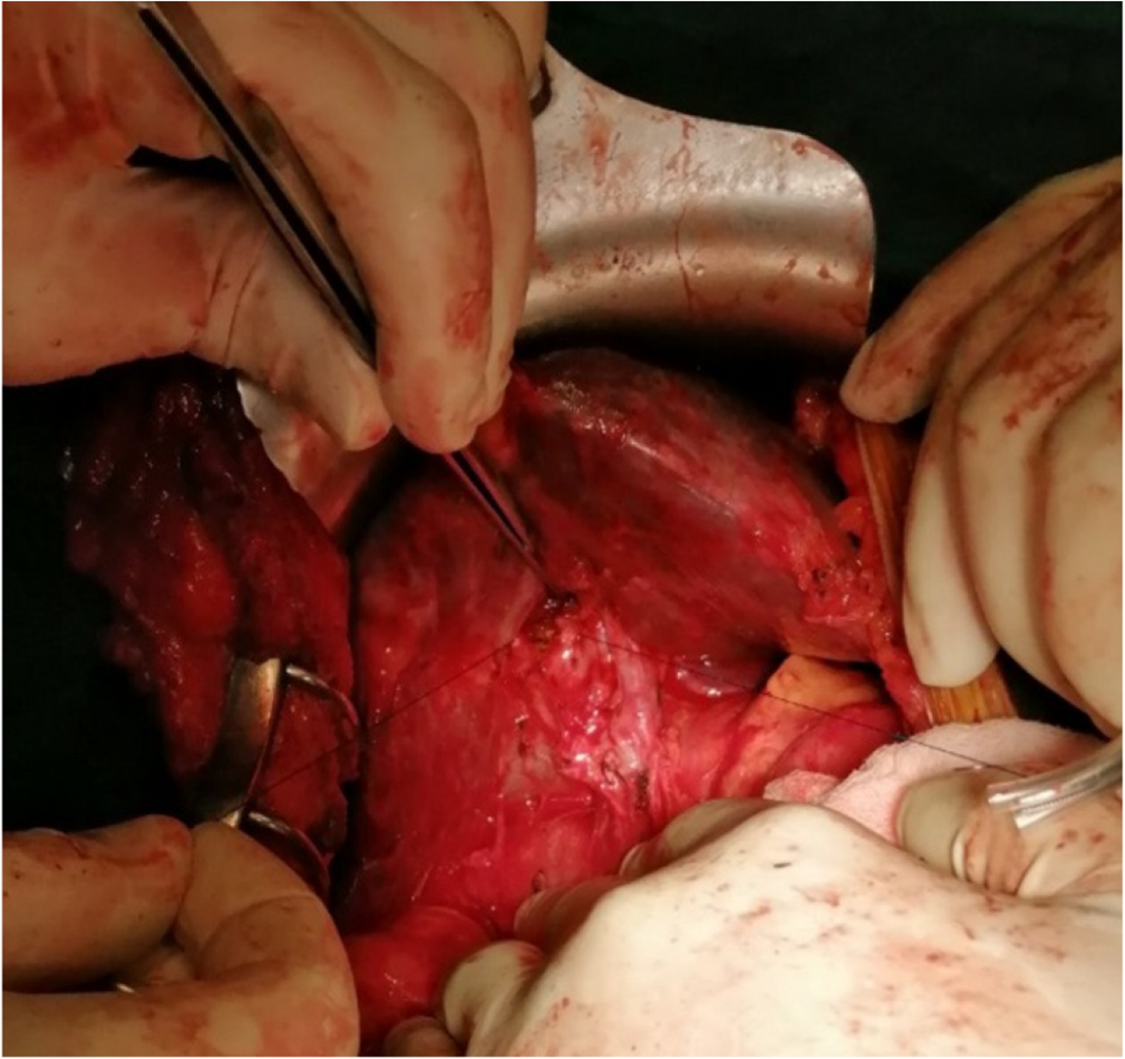
Intraoperative picture of common hepatic duct stump before anastomosis

**Fig. 4:**
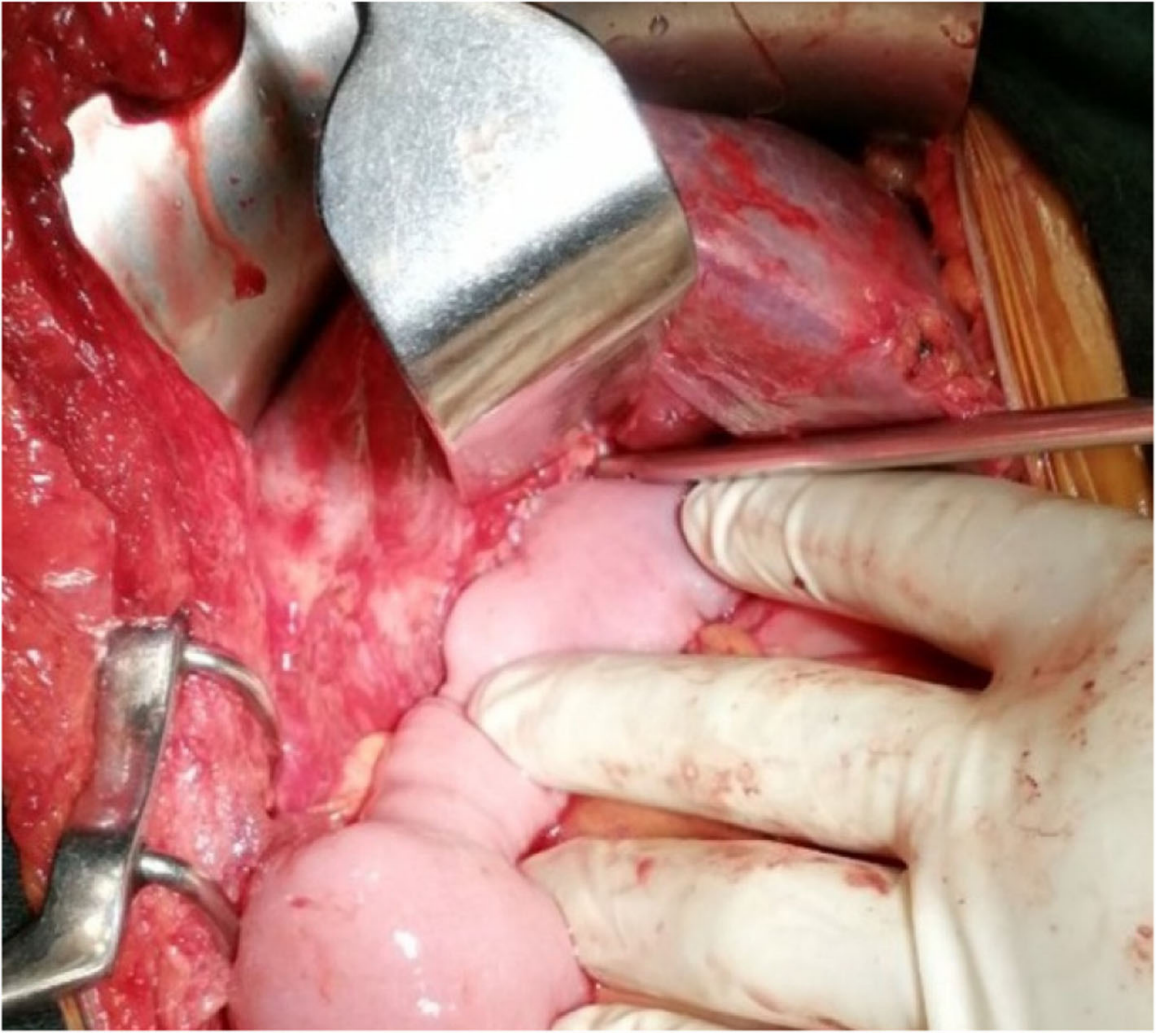
Intraoperative picture of completed hepaticojejunostomy

**Fig. 5:**
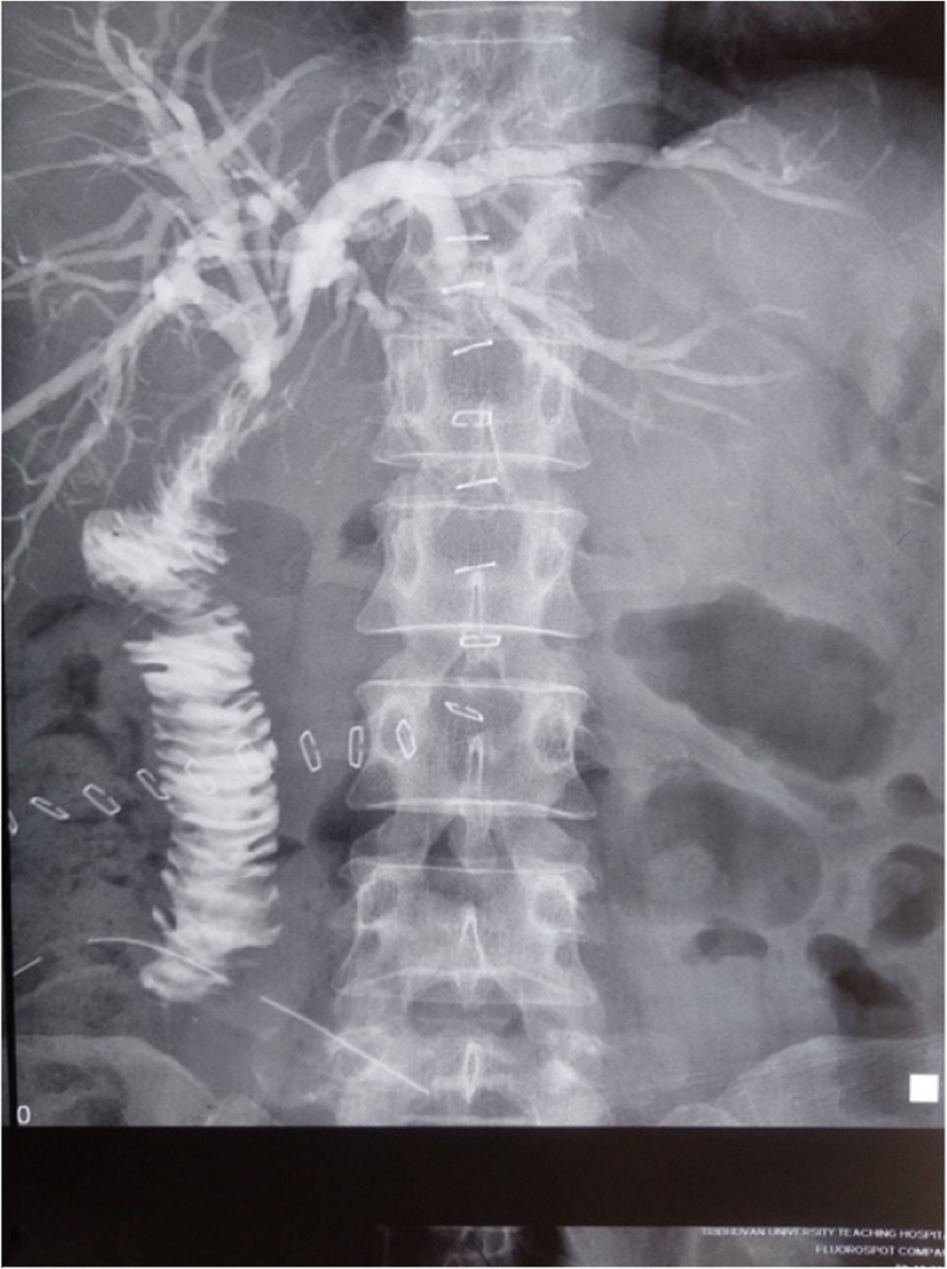
Postoperative tube cholangiogram showing the well-functioning hepaticojejunostomy

The operative findings were dense adhesions with 15-mm-long remnant CHD (Fig. 3) and small hepatoduodenal fistula, and the liver was grossly normal. A Roux-en-Y hepaticojejunostomy (HJ) was made in a single-layer duct to mucosa in a continuous manner using polypropylene 5-0 suture (Figs. 3 and 4). The postoperative period was uneventful with a normal T-tube cholangiogram (Fig. 5), following which the abdominal drain was removed, and the patient was discharged on the sixth postoperative day. Six weeks after surgery, the T-tube cholangiogram was repeated, which showed a well-functioning HJ, and the tube was removed with normal liver function tests. The patient was doing fine through 10 months of follow-up.

## Discussion

Laparoscopic cholecystectomy (LC) is one of the most frequently performed procedures in general and gastrointestinal surgery, and it is now the gold standard treatment for gallstone diseases [2]. Bile duct injury (BDI) is the most dramatic complication after cholecystectomy, showing an increased incidence after introduction of the laparoscopic procedure (0.2–0.4%) [5]. Complete occlusion resulting in major injuries, biliary leakage, and bile duct stricture is a severe long-term complication after LC besides minor bile leakage. Various factors, such as complexity and location of the injury, the degree of inflammation, the presence of ongoing infection or sepsis, and the expertise of the surgeon, will determine the successful management of BDI [6].

We managed our patient, a 55-year-old women with BBS who presented 11 years after complicated cholecystectomy, with Roux-en-Y HJ. She was aymptomatic until 1 month following the index cholecystectomy, when she presented with cholangitis. The asymptomatic period could be explained by the presence of a hepatoduodenal fistula due to repeated inflammation and closure of the fistula and ultimately led to biliary obstruction.

A patient can be asymptomatic for a long time. However, symptomatic patients can present with pain, jaundice, pruritis, recurrent cholangitis, or simply alteration in liver function tests. Dilation of the intrahepatic biliary tree or of the CBD can be found on an ultrasound, but it is not always present. MRCP can accurately delineate the biliary anatomy and the site and length of the stenosis, and it is most useful before planning further treatment. ERCP has been advocated because it can be therapeutic, too. It may be possible to reserve surgery only for patients in whom endoscopic treatment has failed. The endoscopic treatment and new stent materials can offer results similar to those of surgical treatment. Perhaps a rendezvous method in the setting of failure of an ERCP and a successful percutaneous transhepatic cholangiography (PTC) can offer the possibility of dilation and stenting, but there will be a chance of re-stricture and need of multiple interventions. So, considering the treatment options, availability, and technically demanding nature of endoscopic treatment, along with the need for repeated interventions, failure rate, costs, and geographical difficulties, one-time definitive treatment in the form of surgery is preferred [4]. The use of an endoscopic stent as definitive management of BDI is not recommended [7]. BBS should be repaired electively by a Roux-en-Y HJ [7].

The median duration of stricture was 7 months in a study by Sikora *et al.* [8]; the majority of BBS cases present after months to years. As the duration increases, the majority develop complications in the form of portal hypertension or SBC. The longest duration of stricture development following cholecystectomy is difficult to say, but in our patient, it was 11 years.

Biliary strictures are grouped on the basis of the level at which healthy biliary tissue is available for surgical repair as per the Bismuth classification [9]. The Bismuth classification was proposed in an era prior to LC and is related to bile duct strictures. This system is relatively simple to use and is based on how proximally the injury occurs and the length of the healthy proximal bile duct stump. It is an excellent classification in terms of outcome of BDI and for planning the operative repair. However, it does not include any associated vascular injury and does not take into consideration the entire spectrum of injuries. Type I strictures occur in the common bile or hepatic duct at least 2 cm distal to the hilum, whereas type II strictures occur within 2 cm of the hilum. Type III strictures extend to the hilum but with intact confluence, whereas type IV strictures involve the confluence of the right and left hepatic ducts. Type V strictures include the criteria of types I–III strictures as well as an isolated stricture of the right hepatic duct. This classification system has been found to correlate with the outcome of patients following surgical repair [9]. This classification has been universally adopted for use in the classification of biliary injury, especially in complete transection of the bile duct, and it served as the basis for the widely used Strasberg classification [10].

The Strasberg classification is probably the most widely accepted system and builds on the Bismuth classification. It defines major duct injuries as well as the causes of bile leaks. It is relatively simple to remember and use. It is divided into five classes (A–E), with five subclasses in E (E1–E5). Class E is a complete transection of the major bile duct, and the five subtypes (E1–E5) relate to the stump length remaining. It appears that long-term results can be correlated with the Strasberg grade of injury.

The choice of surgical approach should be tailored to the height and extent of the lesion. When the stricture is below the confluence (Bismuth type I or II), a direct anastomosis to the hepatic duct stump is usually straightforward. By contrast, when the stricture encroaches on the confluence of the right and left hepatic ducts (type III) or extends proximally so as to isolate the ducts (type IV), the problem becomes more complex, and good results are more difficult to achieve. In type V, sufficient exposure of both the right and left ducts and the injured aberrant right duct is required for adequate repair. Surgery is associated with better outcome in terms of morbidity, mortality, quality of life, and improved survival [5]. Early identification and prompt management of BBS can prevent devastating sequelae such as recurrent cholangitis and end-stage liver disease needing transplant. Long-term follow-up is essential after the repair of a BBS, because recurrence can develop several years after repair. However, recurrent BBS is best treated with endoscopic balloon dilation [7].

## Conclusion

Iatrogenic injury to the CBD following LC is a rare but devastating complication of one of the most common surgical procedures. BBS can present even after decades and without development of SBC. Benign biliary stricture should be repaired electively by an experienced biliary surgeon by performing a Roux-en-Y HJ.

## Data Availability

The datasets during the current study available from the corresponding author on reasonable request.

## Abbreviations

BBS: Benign biliary stricture
BDI: Bile duct injury
CBD: Common bile duct
CHD: Common hepatic duct
ERCP: Endoscopic retrograde cholangiopancreatography
HJ: Hepaticojejunostomy
LC: Laparoscopic cholecystectomy
MRCP: Magnetic resonance cholangiopancreatography
SBC: Secondary biliary cirrhosis
